# Pharmaceuticals, Pesticides, and Poly- and Perfluoroalkyl Substances at Surface Water Occurrence Levels—Impact of Compound Specific Physicochemical Properties on Nanofiltration and Reverse Osmosis Processes

**DOI:** 10.3390/membranes15120358

**Published:** 2025-11-27

**Authors:** Jelena Šurlan, Claudia F. Galinha, Nikola Maravić, Carla Brazinha, Igor Antić, Jelena Živančev, Nataša Đurišić-Mladenović, Zita Šereš, João G. Crespo

**Affiliations:** 1 Faculty of Technology Novi Sad, University of Novi Sad, Bul. Cara Lazara 1, 21000 Novi Sad, Serbia; 2 LAQV-REQUIMTE, Department of Chemistry, NOVA School of Science and Technology, FCT NOVA, Unversidade NOVA de Lisboa, 2829-516 Caparica, Portugal; 3 Department of Bioengineering and iBB—Institute for Bioengineering and Biosciences, Instituto Superior Técnico, Universidade de Lisboa, 1049-001 Lisboa, Portugal; 4 Associate Laboratory i4HB—Institute for Health and Bioeconomy, Instituto Superior Técnico, Universidade de Lisboa, 1049-001 Lisboa, Portugal; 5 Instituto de Tecnologia Química e Biológica António Xavier, Universidade Nova de Lisboa, Av. da República, 2780-157 Oeiras, Portugal

**Keywords:** water treatment, pharmaceuticals, pesticides, PFAS, nanofiltration, reverse osmosis

## Abstract

Pharmaceutically active compounds (PhACs), pesticides, and poly- and perfluoroalkyl substances (PFAS) are increasingly detected in surface waters at trace concentrations, raising concerns for both aquatic systems and, consequently, human health. Conventional solutions are insufficient to achieve complete removal at trace compound concentrations, highlighting the need for advanced separation technologies. This study aims to comprehensively analyze rejection and removal mechanisms of selected PhACs, pesticides, and PFAS present in water solutions at reported environmentally relevant concentrations (300 ng L^−1^), using two nanofiltration (NF) and one reverse osmosis (RO) polyamide membrane. PhACs, pesticides, and PFAS were selected to cover a broad range of physicochemical properties, specifically molecular mass (MM), dissociation constant (pKa), and octanol–water partition coefficient (logK_o/w_). Rejection values ranged from 42.1% (acetaminophen) to apparent 100% (for multiple compounds), depending on water pH, solute properties, and membrane characteristics. Size exclusion and electrostatic interactions were identified as the primary removal mechanisms, with hydrophobic interactions having a lower impact, particularly for carbamazepine, bezafibrate, and perfluorooctane sulfonic acid (PFOS). Addition of sodium chloride (3 g L^−1^) decreased rejection of most negatively charged compounds due to suppression of membrane surface charge, although clarithromycin and ofloxacin exhibited improved rejection. Presented results provide fundamental insight into compound-specific membrane rejection and highlight the importance of membrane–solute interactions under environmentally realistic conditions. The results support further optimization of NF and RO for targeted compound rejection and provide a baseline for data-driven membrane process modeling.

## 1. Introduction

Contaminants of emerging concern (CECs), such as PhACs, pesticides, and PFAS, are ubiquitously present in the environment with many of them extremely persistent, presenting a major challenge to human health and aquatic fauna [1,2,3]. Conventional wastewater treatment processes, including flocculation, sedimentation, micro- and ultrafiltration, and disinfection, are inefficient for complete CECs removal, and thus wastewater discharge is among the primary sources of numerous CECs in aquatic systems. On the other hand, advanced technologies, such as NF and RO, are often considered for achieving efficient removal of micropollutants [4,5,6,7]. Compared to RO membranes that, due to the small pore size (≈0.25 nm), reject most ions (≈0.25 nm), NF membranes have higher pore size (0.5–2 nm), permeability, and selectivity towards divalent and polyvalent ions, while allowing passage of monovalent ions and small molecules [8]. Both NF and RO membranes are widely studied in water treatment processes due to the ability to remove different pollutants with low molecular mass (MM), including some CECs [9,10]. CECs removal using NF and RO is dependent on multiple factors, including operating parameters, solution properties, membrane characteristics, and CECs physicochemical properties [9,11,12,13,14].

Multiple studies have reported on removal of PhACs, pesticides, and PFAS from various water samples [10,11,13,14,15,16,17,18,19,20,21,22,23,24]; however, most of the reviewed studies focused on the influence of operating parameters, solution properties, and membrane characteristics on CECs removal. Detailed research focusing on specific correlations between individual CECs’ physicochemical properties and membrane rejection is still scarce. In particular, it remains unclear which molecular descriptor most reliably predicts and affects solute–membrane interactions across different membrane types and operating conditions [25].

Understanding the correlation between main CECs properties (MM, pKa, and logK_o/w_) and membrane rejection at different operating conditions can provide valuable information regarding membrane process optimization for specific CECs removal. Furthermore, most of the reviewed studies focus on higher concentrations of CECs compared to occurrence concentrations reported in surface waters (pg/L-µg/L) [3]. Therefore, there is a critical need for studies that not only investigate how CECs’ specific molecular descriptors govern membrane rejection but also evaluate CECs removal at environmentally relevant concentrations.

The aim of this study is to evaluate the potential of selected NF and RO membranes for the removal of target PhACs, pesticides, and PFAS representatives having different physicochemical properties at environmentally relevant concentrations in water samples and provide a detailed insight into the relation between individual CECs’ molecular descriptors and membrane rejection at different operating conditions. This study employed ultrapure water spiked with selected CECs at matching environmental occurrence levels as a model matrix to allow controlled, reproducible conditions and to isolate the effects of target compounds on NF/RO rejection performance without the variability introduced by natural organic matter, colloids, or other co-contaminants. This work was performed at concentrations representative of real-world occurrence levels [26,27,28]. The primary aim was to investigate fundamental removal mechanisms under well-defined conditions before introducing additional complexity. Future work will extend these investigations to environmentally relevant matrices, incorporating natural organic matter and particulate matter to evaluate fouling behavior, long-term performance, and scalability under realistic conditions. Discussion concerning permeate fluxes obtained during the process was excluded in order to focus on identifying relevant solute removal mechanisms. Furthermore, impact of sodium chloride presence in water samples was evaluated in experiments where RO membrane was used, since high levels of salts are often present in various water bodies, due to saltwater intrusion, climate change impact (droughts), evaporation in closed basins, industrial discharges with high salt concentration, etc. The findings of this study contribute to a better understanding of the key factors influencing PhACs, pesticides, and PFAS removal by membrane-based processes, providing valuable insights for optimizing treatment processes and improving water quality management.

## 2. Materials and Methods

### 2.1. Chemicals and Reagents

Fourteen compounds were initially selected for investigation; however, the final results on the efficiency of membrane processes are presented for twelve compounds due to limitations of the applied analytical method, as explained later. Six PhACs, four pesticides, and four PFAS were initially selected as CECs representatives based on their differences in physicochemical properties, more specifically MM, pKa, and logK_o/w_ (Table 1). MM of selected CECs ranges from 151.2 Da to 748.0 Da. Nine selected CECs are considered hydrophobic (logK_o/w_ > 2) and five CECs are classified as hydrophilic (log K_o/w_ < 2), based on the logK_o/w_ value limit [29]. Based on their pKa value, four CECs are positively charged, two are neutral, and eight are negatively charged at pH 7.

Samples were prepared using high-purity CECs standards from Sigma Aldrich (St. Louis, MO, USA) or LGC (Augsburg, Germany). Purities of the used analytical standards were as follows: clarithromycin (99%), ofloxacin (>95%), carbamazepine (100%), acetaminophen (>99%), salbutamol (100%), bezafibrate (99%), carbofuran (>99%), acetamiprid (>99%), malathion (>99%), propiconazole (>98%), PFOSA (>97%), PFOS (100%), PFBA (100%), and PFBS (>99%). Stock solution with targeted PhACs, pesticides, and PFAS was prepared using LC-MS grade methanol (MeOH, ≥99.9%) (VWR International, Radnor, PA, USA). Ultrapure (Milli Q) water (18 MΩ cm) (Advantage system from Millipore, Molsheim, France), was used for model water (feed) preparation. Diluted sodium hydroxide (>98%) and hydrochloric acid (>36.5%) solutions (concentration 0.1 mol L^−1^) were used to set solution pH value at pH 4, pH 7, and pH 10. Sodium chloride (>99.5%) was added in RO feed solution at pH 7 in a concentration relevant in brackish water treatment (3 g L^−1^).

### 2.2. NF and RO Experiments

Feed solutions were prepared in Milli Q water by spiking the water with solution containing PhACs, pesticides, and PFAS (concentration of each individual CEC in each feed solution was 300 ng L^−1^), followed by pH adjustment and sodium chloride addition. The target concentration of 300 ng L^−1^ for each compound in the spiked model mixture was selected based on concentrations previously reported for CECs in surface water and treated wastewater effluents. Numerous monitoring studies have documented CECs occurrence in the low ng/L to low μg/L range, with median or mean concentrations for many pharmaceuticals and personal care products frequently falling between 100 and 500 ng/L [26,27,28,29,30].

Three thin-film composite (TFC) polyamide membranes (SW30HR, Desal-5 DK, and NF270) were selected based on the differences in molecular weight cut off (MWCO), according to the manufacturers’ specifications. Relevant characteristics of the selected membranes are shown in Table 2.

Experiments were conducted in a METCell dead-end stirred cell unit from Evonik Industries AG (Essen, Germany) (Figure S1 in Supplementary Material ). The active surface area of the membrane is 51.4 cm^2^. Prior to conducting the experiments, membranes were soaked in ultrapure water for 24 h. High pressure nitrogen gas cylinder with a pressure regulator was used to adjust the pressure in the feed compartment. Prior to the addition of feed solution, membranes were compacted in the cell unit with ultrapure water until a constant flux was obtained. Pressure for each membrane was chosen based on the flow of the ultrapure water through the pristine membrane, in order to achieve similar duration of each experiment. Pressure was set based on the membrane permeate flow at 3 bar, 10 bar, and 30 bar for NF270, Desal-5 DK, and SW30HR membranes, respectively, and was kept constant throughout the experiments. Feed solutions were stirred at 450 rpm for the duration of the experiments. This vigorous stirring ensured that no external mass transfer limitations occur (experimentally validated). Membrane flux was monitored for the duration of the experiments by acquisition of the permeate mass with an accuracy of 0.01 g. Permeate flow, *Q_p_* (L h^−1^), and permeate flux, *J* (L h^−1^ cm^−2^), were calculated using the following equations, respectively:

(1) $$Q_{p}=\;\frac{V}{t}$$

(2) $$J=\frac{Q_{p}}{A}$$

where *V* (L) and *t* (h) represent permeate volume and time of permeate collection, respectively, whereas *A* represents the membrane surface area (cm^2^).

The experiments lasted until the first 100 mL of permeate were collected, with a remaining 100 mL of retentate. Concentrations of targeted PhACs, pesticides, and PFAS were analyzed in the feed, permeate, and retentate solutions.

Apparent rejections, *R* (%), were calculated using the following equation:

(3) $$R=\left(\frac{c_{r}}{c_{r}+c_{p}}\right)*100$$

where *c_r_* and *c_p_* represent CECs concentration (ng mL^−1^) in retentate (concentration in the feed compartment in each instant) and permeate, respectively. Results and discussion of membrane adsorption were not included in the corresponding article, since adsorbed CECs amounts to the membrane surface are below the limit of quantification for UHPLC-MS/MS, due to low, but environmentally relevant, initial CECs concentration. Therefore, Equation (3) was used in order to more clearly evaluate rejection and removal mechanisms without the interference of possible adsorption onto membrane surface. Specific values of measured data are provided in metadata file. Rejection values presented in Section 3 are shown without errors and standard deviation values; however, charts with errors are shown in Figure S1 in Supplementary Material. In experiments where *c_p_* was below the limit of detection, *c_p_* was considered as a non-detectable concentration, and the calculated R value was assumed to be 100%.

### 2.3. Sample Preparation and Instrumental Analysis

Sample preparation was carried out using the previously described method in a study published by Petrović et al. (2014) [34]. Briefly, preparation of collected sample solutions (feed, permeate, and retentate) for instrumental analysis included solid phase extraction (SPE). SPE was performed by using single-layer sorbents (Oasis HLB, 200 mg, 6 mL, Waters Corporation, Milford, MA, USA). HLB sorbents were selected based on their hydrophilic–lipophilic nature, which enables extraction of a wide range of CECs with different physicochemical properties. Cartridges were positioned in a vacuum manifold and conditioned by 5 mL of MeOH and 5 mL of ultrapure water, followed by loading the sample and rinsing with 5 mL of ultrapure water. Afterwards, sorbents were dried for 2 h by passing air through the sorbent. Targeted CECs were eluted from the sorbents with MeOH (2 × 4 mL). Obtained eluates were evaporated to dryness at 30 °C under a nitrogen stream and reconstituted in 1 mL of first gradient mobile phase. High-performance liquid chromatography coupled with triple quadrupole mass spectrometry, UHPLC-MS/MS (Thermo Fisher Scientific, Waltham, MA, USA), was used for instrumental analysis of selected CECs, which is described in detail in a study published by Rakić et al. (2023) [35]. Conditions and methodology for instrumental analysis are described in detail in Supplementary Material.

Recoveries determined for all 14 initially selected compounds are presented in Supplementary Material (Table S3). Although four PFAS were included in model feed solutions, rejection values for two of them, perfluorooctane sulfonamide (PFOSA) and perfluorobutanoic acid (PFBA), were not considered in this study due to the very low recoveries. Specifically, mean recoveries were 7.36% for PFOSA and 20.92% for PFBA (Table S3 in Supplementary Material), which are considered insufficient for reliable quantification and, consequently, for robust calculation of rejection. Hydrophilic–lipophilic balance (HLB) polymeric sorbents, due to their “dual nature”, can be applied for PFAS extraction; however, low recoveries could be expected for short-chain perfluoroalkyl carboxylic acids [36], e.g., PFBA, due to inefficient retention onto the sorbent [37]. This behavior is in agreement with previous reports showing poor HLB retention and low recoveries for short-chain PFCAs, including PFBA [38,39]. Additionally, PFOSA exhibits pH-dependent extraction behavior, with optimal recoveries at pH 8 [40], whereas in this work, pH 7 was selected as a compromise condition to enable simultaneous extraction of CECs with diverse physicochemical properties. For these reasons, PFOSA and PFBA were excluded from the rejection evaluation.

Additionally, lower recovery was observed for ofloxacin (~46%), probably due to its high hydrophilicity, which may hinder efficient extraction by HLB polymeric cartridges. Nevertheless, the results on rejection values for this compound are presented here, as it is assumed that the same recovery rate applies to this compound in both *c_r_* and *c_p_* samples. Consequently, the percentage ratio calculated using Equation (3) remains unaffected, providing valuable insights into ofloxacin removal.

According to Gros et al. (2012) [41], it is challenging to find optimal conditions for each target analyte in multi-residue methodologies; therefore, it is necessary to select optimal conditions for the highest number of compounds. Recoveries of all selected CECs are given in the Supplementary Material.

## 3. Results and Discussion

Three polyamide membranes with different MWCO (SW30HR, Desal-5 DK, and NF270) were selected for thorough analysis of CECs rejections at different operating pH values. Twelve CECs, including six PhACs, four pesticides, and two PFAS, were selected in order to cover a wide range of molecular descriptors (MM, pKa, and logK_o/w_) and evaluate the impact of each descriptor on solute rejection and associated mechanisms. Table 1 and Table 2 summarize CECs’ physicochemical properties and selected membranes’ properties, respectively.

### 3.1. Impact of CECs’ Physicochemical Properties and Operating pH on CECs Rejection

**Figure 1:**
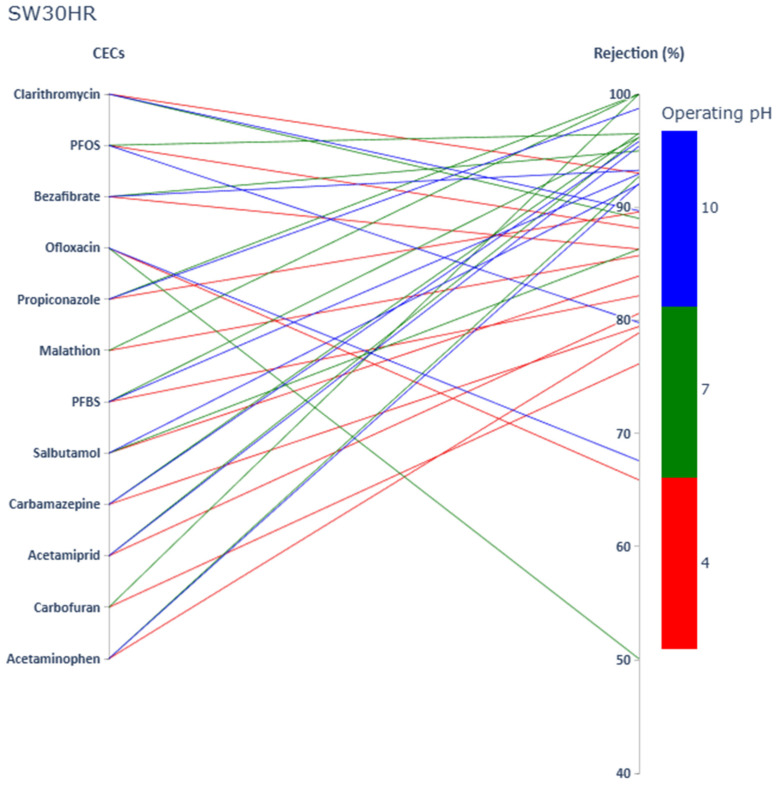
PhACs, pesticides, and PFAS rejection values (sorted by increasing MM) at pH 4, pH 7, and pH 10 by using the SW30HR membrane.

**Figure 2:**
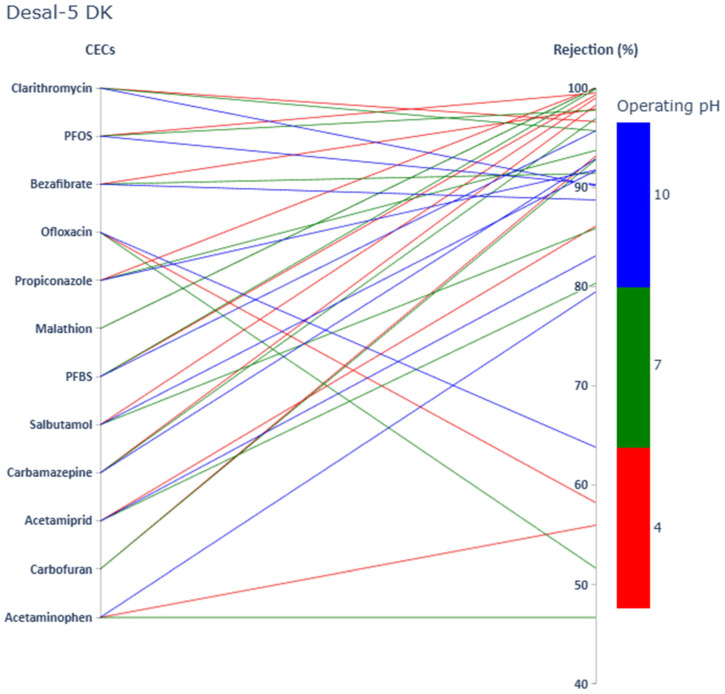
PhACs, pesticides, and PFAS rejection values (sorted by increasing MM) at pH 4, pH 7, and pH 10 by using the Desal-5 DK membrane.

**Figure 3:**
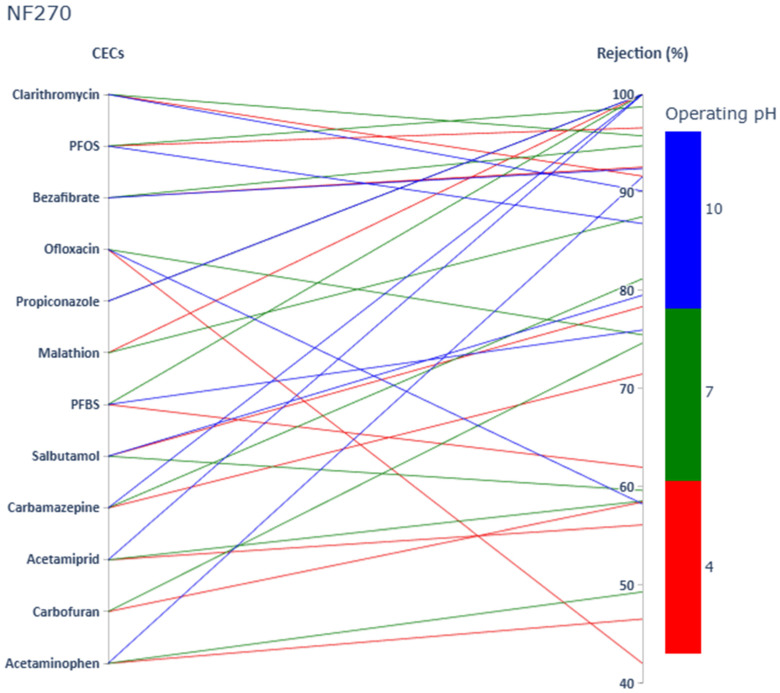
PhACs, pesticides, and PFAS rejection values (sorted by increasing MM) at pH 4, pH 7, and pH 10 by using the NF270 membrane.

Rejection values of twelve targeted CECs (including two PFAS, four pesticides, and six PhACs) at three pH values by using SW30HR, Desal-5 DK, and NF270 membranes are shown in Figure 1, Figure 2 and Figure 3, respectively.

Rejection values ranged from 42.1% to apparent 100% by selected membranes. Rejection values of the same compound varied greatly depending on selected membrane and operating pH. Figure 1, Figure 2 and Figure 3 allow, by a simple visual observation, a confirmation of the relevance of size exclusion on the rejection of the selected solutes. It seems clear that there is a general trend confirming that the tighter the membrane, the higher the solute rejection (sequence SW30HR, Desal-5 DK, and NF270) as previously reported in numerous studies [42,43]. However, at pH 4, the observed rejection pattern did not fully conform to this expected trend. Also, from these figures it can easily be concluded that, in general, at higher pH, where the membranes under study are negatively charged, as well as the selected solutes, rejection is higher due to charge repulsion, confirming the relevance of electrostatic mechanisms in solute rejection.

It should also be noticed that when using the SW30HR membrane, the lowest rejection values regarding all tested PhACs, pesticides, and PFAS were obtained at pH 4. Even though differences in CECs’ molecular descriptors were significant in terms of MM, pKa, and logK_o/w_, rejection values remained low for all samples at pH 4. Therefore, it can be assumed that lower rejection values obtained were caused by changes in the SW30HR membrane. Polyamide membranes are extremely sensitive to external factors (such as operating pH), which could influence the membrane swelling, therefore increasing the space between the membrane fibers and increasing permeation of CECs through the membrane. However, further research focused on SW30HR properties is required to determine the exact cause for lower performance of SW30HR membrane at pH 4.

In the following sections, rejection values and removal mechanisms of each individual PhAC, pesticide, and PFAS, with the three selected membranes and three operating pH values, are thoroughly discussed, focusing on CECs’ specific properties presented in Table 1 (MM, pKa, and logK_o/w_)_._

#### 3.1.1. Acetaminophen

Rejection values of acetaminophen at pH 4 decreased with an increase in MWCO of the membrane (SW30HR > Desal-5 DK > NF270). Relatively low rejection values of acetaminophen (MM = 151.2 Da) by Desal-5 DK and NF270 membranes (MWCO 150–300 Da and 400 Da, respectively), in addition to uncharged membrane surfaces at pH 4 (Table 2), suggest size exclusion as the main removal mechanism at pH 4. Increase in operating pH to pH 7 decreased acetaminophen rejection value when using the Desal-5 DK membrane, whereas the rejection of acetaminophen increased by 13.8% when the SW30HR membrane was used. As mentioned previously in this section, the SW30HR membrane has an overall lower performance at pH 4 for all selected CECs; therefore, increase in rejection with an increase in pH value could be expected. However, an increase in operating pH to pH 7 led to a negative charge of the Desal-5 DK membrane surface, while the acetaminophen molecule keeps a positive charge (pKa > pH). Therefore, decrease in rejection value due to electrostatic attraction between acetaminophen and membrane surface could occur. Further increase in pH value to 10 resulted in an evident increase in rejection of acetaminophen by all three membranes (R > 78%). Hence, electrostatic repulsion between the negatively charged membrane surfaces and negatively charged acetaminophen (pKa < pH) contributed to increased rejection values. The greatest increase in rejection of acetaminophen with increase in operating pH (from 4 to 10) was observed when the NF270 membrane was used. Increase in rejection of acetaminophen by NF membranes with an increase in pH value was also observed in a previously published study [44]. Size exclusion and electrostatic interactions could be considered as the most important removal mechanisms by selected membranes, whereas, due to the acetaminophen hydrophilic nature, hydrophobic interactions had no noticeable impact on rejection.

#### 3.1.2. Carbamazepine

Overall, high rejection values of carbamazepine (MM 236.3 Da), above 71%, were observed by all three membranes at pH 4. The lowest rejection of carbamazepine at pH 4 was observed when using the NF270 membrane, whereas the highest rejection of carbamazepine was observed by the Desal-5 DK membrane, suggesting size exclusion as relevant mechanism. Carbamazepine rejection by SW30HR membrane at pH 4 could be influenced by the changes on the membrane surface, as mentioned previously in this section; therefore, rejection by SW30HR membrane is lower compared to Desal-5 DK, even with lower MWCO. Zero net surface charge of SW30HR, Desal-5 DK, and NF270 membranes at pH 4 excluded electrostatic interactions as removal mechanism. However, the hydrophobic nature of carbamazepine could introduce some impact of hydrophobic interactions on rejection. Increased compound hydrophobicity might lead to an increase in adsorption on the membrane surface, therefore increasing the permeation through the membrane, leading to lower rejections and higher compound concentrations detected in the permeate [11].

Further increase in pH increased carbamazepine rejection values by the selected membranes to values above 81% and 92% at pH 7 and pH 10, respectively. Carbamazepine is negatively charged at all tested pH values (pKa = 2.3), whereas SW30HR, Desal-5 DK, and NF270 are negatively charged at pH 7 and pH 10 (Table 2). Increase in carbamazepine rejection with an increase in pH value suggests strong impact of electrostatic repulsion on carbamazepine rejection. The most evident influence of increasing pH value on carbamazepine rejection was observed when the NF270 membrane was used. An increase in pH value increases the NF270 membrane negative surface charge [45]. Additionally, due to the high MWCO, impact of electrostatic repulsion on rejection would be most evident when using the NF270 membrane.

#### 3.1.3. Salbutamol

Salbutamol rejection values above 78% were observed at pH 4 by all three membranes. However, with an increase in pH value (from 4 to 7) and use of the Desal-5 DK and NF270 membranes, rejection values decreased. Salbutamol is positively charged at pH 7 (pKa > pH), whereas the membrane surfaces are negatively charged at pH 7, and therefore electrostatic attraction could hinder the efficiency of NF process. Higher concentrations of salbutamol across the membrane surface due to electrostatic attraction could increase the permeation of salbutamol through the membrane and, consequently, lower rejection values. Salbutamol rejection values at pH 4 and pH 7 when using the SW30HR membrane were similar; however, the MM of salbutamol (239.3 Da) is clearly higher than the SW30HR MWCO (100 Da). Therefore, salbutamol rejection by the SW30HR membrane could mostly be attributed to size exclusion. Rejection values of salbutamol above 90% were noticed at pH 10 when the SW30HR and the Desal-5 DK membranes were used, whereas a rejection of 79.54% was observed in NF270 experiments. The neutral form of salbutamol at pH 10 would exclude electrostatic interactions as a removal mechanism. Furthermore, salbutamol is considered hydrophilic (logK_o/w_ < 2); therefore, hydrophobic interactions should not impact the overall rejection by the selected membranes. Therefore, size exclusion is considered the most dominant removal mechanism acting at pH 10.

Similar MM of salbutamol and carbamazepine suggest that size exclusion is not the sole removal mechanism; therefore, differences in their rejection values might be explained by other properties, such as electrical charge (according to their pKa) and hydrophobic interactions (according to their logK_o/w_ values). Based on the logK_o/w_ value, carbamazepine is considered hydrophobic and salbutamol hydrophilic. Furthermore, carbamazepine is negatively charged at all tested pH values, whereas salbutamol is positively charged at pH 4 and pH 7 and neutral at pH 10. Zero net membrane surface charge would exclude electrostatic interactions at pH 4; however, the hydrophobic nature of carbamazepine could explain the slightly lower rejection value in comparison to salbutamol. At higher pH values, carbamazepine had higher rejection values compared to salbutamol, due to impact of electrostatic repulsion.

#### 3.1.4. Ofloxacin

Overall rejections of ofloxacin were clearly lower (below 68%) compared to other solutes. High rejections of ofloxacin at neutral pH values by using different membranes were achieved by other authors [46,47,48]; therefore, higher rejection values could be expected. Even though the rejection values of ofloxacin were lower overall, comparison between experiments at different pH values, along with the corresponding impact of ofloxacin’s molecular characteristics, were still relevant, since the low recovery in sample preparation (discussed in Section 2.3) applied for all obtained samples. Ofloxacin rejection values increased with a decrease in membrane MWCO at all operating pH values, as expected. The lowest rejection of ofloxacin by all three membranes was observed at pH 4 and pH 7. It can be assumed that electrostatic interactions were not present due to the zero net surface charge at pH 4 and the zwitterion form of ofloxacin at pH 7 (pKa_1_ < pH < pKa_2_); therefore, the only relevant removal mechanism was size exclusion. The highest rejection values of ofloxacin were observed at pH 10 due to the additional mechanism of electrostatic repulsion between the negatively charged ofloxacin and the negatively charged membrane surface.

#### 3.1.5. Bezafibrate

Rejection values of bezafibrate in all experiments were above 85%. The relatively high MM of bezafibrate (361.8 Da) compared to other selected CECs explains the overall high rejection values due to a size exclusion mechanism. The lowest rejection of bezafibrate at pH 4 obtained with the SW30HR membrane suggests an influence of the previously discussed changes in the RO membrane at low pH values. Additionally, the hydrophobic nature of bezafibrate (logK_o/w_ > 2) might also lead to lower rejection values obtained at pH 4, similar to that previously discussed for carbamazepine. Increased hydrophobicity of the compound could result in a decreased rejection by the membrane due to the hydrophobic interactions between the membrane and the targeted compound [11]. Bezafibrate is negatively charged at all tested pH values (pKa < pH); however, electrostatic repulsion impact on rejection values was not as dominant a removal mechanism as size exclusion due to the high MM of bezafibrate.

#### 3.1.6. Clarithromycin

Clarithromycin rejection values above 89% were observed in all experiments. High rejection values of clarithromycin could be attributed to size exclusion, since the MM of clarithromycin (748.0 Da) is significantly higher than the MWCO of all three membranes (Table 2). The positive charge of clarithromycin at pH 7 (pKa > pH) might indicate electrostatic attraction between clarithromycin and the membranes’ surface; however, the high MM of clarithromycin suggests that size exclusion has a greater impact on the rejection of clarithromycin by the selected membranes. Additionally, the impact of clarithromycin’s hydrophobic nature on rejection was not as evident; therefore, size exclusion might be considered the main removal mechanism of clarithromycin.

#### 3.1.7. Carbofuran

Carbofuran rejection values ranged from 58.47% to 93.14% in all experiments at pH 4 and pH 7. However, carbofuran was not detected in the permeate in all tested solutions at pH 10. Half-life of carbofuran at pH 10 is 1.2 h in buffered deionized water, due to increased hydrolysis of carbofuran at higher pH values [49], and hence, intense degradation of carbofuran could occur in sample solutions. Therefore, carbofuran was not detected in all samples at pH 10 due to possible degradation caused by increased hydrolysis.

The highest rejection value of carbofuran at pH 4 was observed by using the Desal-5 DK membrane, whereas the lowest rejection value was observed by using the NF270 membrane. Since the MM of carbofuran is 221.3 Da, and Desal-5 DK and NF270 membranes’ MWCO’s are in the range 150–300 Da and 400 Da, respectively, size exclusion could be considered the main removal mechanism at pH 4. However, lower rejections of carbofuran observed by the SW30HR membrane at pH 4 indicate an influence of RO membrane changes, as discussed above. Increase in carbofuran rejection by SW30HR and NF270 membranes with an increase in pH from pH 4 to pH 7 was observed. Since carbofuran is positively charged at pH 7 and membranes are negatively charged, a negative influence of electrostatic attraction between carbofuran and the membrane surface on rejection could be expected; however, the opposite occurred. Influence of other CECs’ physicochemical properties currently not included in this study, such as compounds’ geometry, solubility, etc., could also influence the rejection; however, further research is required to assess possible impact of other properties on rejection.

#### 3.1.8. Acetamiprid

Rejection values above 80% were observed at all pH values by using the SW30HR and the Desal-5 DK membranes. Overall, high acetamiprid rejections by SW30HR and Desal-5 DK membranes could be attributed to size exclusion (MM = 222.7 Da). Changes in rejection of acetamiprid with changes in operating pH by using the Desal-5 DK membrane were not as evident; however, rejection of acetamiprid with the SW30HR membrane increased with an increase in pH value. Acetamiprid is negatively charged at all pH values (pKa < pH); therefore, electrostatic repulsion between negatively charged acetamiprid and negative SW30HR membrane surface at pH 7 and pH 10 could induce higher rejection values. High rejection of acetamiprid (apparent 100%) was observed by using the NF270 membrane at pH 10, compared to pH 4 and pH 7 (56.14% and 58.57%, respectively). Since the NF270 membrane MWCO is clearly higher than the acetamiprid MM, lower rejection values were expected. However, the high increase in rejection at pH 10 might be explained by the impact of electrostatic repulsion under these conditions.

The similar MM of carbofuran and acetamiprid (Table 1) opened the possibility for a comparative evaluation of pKa and logK_o/w_ impact on membrane rejection. Differences in rejections of carbofuran and acetamiprid were not as evident at pH 4 due to the absence of electrostatic interactions with the membrane surface. However, the rejection value for hydrophobic carbofuran with the SW30HR membrane at pH 4 was slightly lower compared to the rejection of hydrophilic acetamiprid, which could be attributed to hydrophobic interactions between carbofuran and SW30HR membrane. Positively charged carbofuran had higher rejections than the negatively charged acetamiprid by all three membranes at pH 7, and therefore it can be assumed that other molecular properties, not included in this study, impacted the rejection values observed. Due to the intense hydrolysis of carbofuran at pH 10, evaluation of differences in rejections of acetamiprid and carbofuran could not be performed.

#### 3.1.9. Malathion

Malathion rejection values above 85% were observed at pH 4 and pH 7. Malathion was not detected in feed, permeate, and retentate solutions at pH 10. Degradation of malathion in water is dependent on pH value and, above pH 7, degradation of malathion is increased due to hydrolysis [50]. Reported half-life of malathion is 0.2 weeks at pH 8. Furthermore, half-life of malathion decreases continuously with an increase in pH of the solution. At pH 10, hydrolysis and degradation of malathion could occur in sample solutions and, therefore, malathion could not be detected.

Rejection values slightly above 85% were observed by using the SW30HR membrane at pH 4 and NF270 membrane at pH 7. In all other experiments, apparent 100% rejections were obtained. The obtained apparent 100% rejections could indicate other effects besides size exclusion mechanism, since the NF270 MWCO value is higher than the malathion MM value. Therefore, other molecular properties of malathion might impact on rejection, such as molecular geometry and dimensions. However, previously discussed changes on the SW30HR membrane surface could also cause a lower rejection at pH 4. Due to the malathion isoelectric point at pH 7 (pKa = pH), electrostatic repulsion between the NF270 membrane surface and malathion is not expected to occur.

#### 3.1.10. Propiconazole

Propiconazole rejection values above 89% were observed in all experiments. Apparent 100% rejection of propiconazole was obtained by NF270 membrane at all pH values. The relatively high MM of propiconazole (MM > MWCO of SW30HR and Desal-5 DK), resulted in high rejection values. Furthermore, the higher MWCO of NF270 membrane (MWCO > MM) did not negatively affect the rejection. Even though overall high rejection values were obtained, the impact of operating pH was observed. The lowest propiconazole rejection value was observed with SW30HR membrane at pH 4 (due to the possible changes on the membrane surface); however, rejection values increased to over 98% at pH 7 and pH 10. Increase in pH value caused a slight decrease in rejection of propiconazole by Desal-5 DK membrane, but the observed rejection values remained above 90%. Therefore, it can be concluded that, besides size exclusion, electrostatic interactions might influence rejection of propiconazole.

Propiconazole and malathion have similar MM; however, their pKa values differ significantly (1.09 and 6.8, respectively). Higher rejection values of propiconazole compared to malathion were observed at pH 7 by the NF270 membrane due to electrostatic repulsion between propiconazole and the negatively charged membrane surface. However, similar propiconazole and malathion rejection values were obtained by all selected membranes at pH 4, due to the absence of electrostatic interactions (the selected membranes’ pKa value is close to pH 4). Due to the short half-life of malathion at pH 10, comparison between malathion and propiconazole rejections at pH 10 was not evaluated.

#### 3.1.11. PFBS

Overall, high rejection values of PFBS (above 82%) were observed in all experiments, with exception of the NF270 membrane (pH 4 and pH 10 experiments). Since the MM of PFBS is 300.1 Da, size exclusion could be considered as the main removal mechanism when the SW30HR and Desal-5 DK membranes were used. Higher rejection values were obtained with an increase in sample pH value when the SW30HR and NF270 membranes were used. The corresponding effect suggests an impact of electrostatic repulsion between the negatively charged membrane surface and the negatively charged PFBS. However, a clear influence of pH value on the rejection of PFBS by the Desal-5 DK membrane could not be observed, since all rejection values were above 95%.

#### 3.1.12. PFOS

PFOS rejection values above 96% were obtained in samples at pH 4 and pH 7 by all selected membranes, with exception of the SW30HR membrane at pH 4 (possible changes in the membrane surface). Therefore, it can be assumed that size exclusion dominates the membrane rejection processes at pH 4 and pH 7 (PFOS MM = 500.1 Da). However, PFOS rejection values by all selected membranes at pH 10 decreased. Since PFOS MM is clearly higher than the MWCO of selected membranes and both PFOS and membrane surfaces are negatively charged (suggesting electrostatic repulsion), higher rejection values were expected. Therefore, membrane rejection of PFOS might be influenced by other properties.

A clear decrease in the perfluorooctanoate (PFOA) rejection value at pH 10 was observed by using the NF90 membrane in a previously published study [51], attributing high concentrations of PFOA in the permeate to membrane swelling. According to authors, at pH 10, the functional groups on the membrane surface dissociate fully and the negative charges of the polymer chain (polyamide) repel each other and increase the membrane free volume with impact on its rejection performance, thus leading to a higher solute permeation. However, if adequate, this explanation should apply to all compounds at pH 10. Therefore, further explanation is required for understanding the decrease observed only for the PFOS rejection value. PFOS has a strong hydrophobic nature (logK_o/w_ = 4.49) due to the presence of a so-called hydrophobic tail, as well as a higher hydrophobicity compared to other selected CECs. According to the manufacturers’ specifications, SW30HR, Desal-5 DK, and NF270 membrane surfaces are polyamide (hydrophilic), whereas the support layer consists of polysulfone, which is considered hydrophobic. Therefore, if membrane swelling occurs due to repelling between negative charges on the membrane surface, solvent and CECs molecules in the mixture can move more freely within the membrane, opening possibility for hydrophobic PFOS to access hydrophobic regions of the membrane (support layer) [52]. However, abovementioned membrane swelling effects had no evident impact on other CECs due to their lower hydrophobicity and stronger impact of electrostatic repulsion on their rejection at pH 10.

Table 3 summarizes the discussion about the dominant removal mechanisms of selected CECs at three pH values by the SW30HR, Desal-5 DK, and NF270 membranes.

Based on the obtained results, size exclusion could be considered as the main removal mechanism of selected PhACs, pesticides, and PFAS at pH 4 by all selected membranes. Since membranes’ isoelectric point values are close to pH 4, electrostatic interactions do not seem to affect the membrane separation process at this pH. However, the hydrophobic nature of carbamazepine, carbofuran, bezafibrate, malathion, and propiconazole seems to have a noticeable negative impact on solute rejection at pH 4, due to hydrophobic interactions with the membrane surface.

In addition to size exclusion, electrostatic interactions between negatively charged membranes and charged CECs influence the rejection of CECs at pH 7. Improved rejections of CECs with negative charge at pH 7 by the selected membranes were observed. However, due to electrostatic attraction between the negatively charged membrane surface and positively charged acetaminophen and salbutamol at pH 7, lower rejection values were observed compared to pH 4 experiments.

The most evident impact of electrostatic interactions between membrane surface and charged PhACs, pesticides, and PFAS was observed at pH 10. Due to electrostatic repulsion between negatively charged membrane surfaces and negatively charged CECs, rejection values of acetaminophen, carbamazepine, ofloxacin, bezafibrate, acetamiprid, propiconazole, and PFBS were higher at pH 10 compared to lower pH values.

The obtained results suggest size exclusion and electrostatic interactions as most relevant removal mechanisms for SW30HR and NF270 membranes. Impact of pH value and, consequently, electrostatic interactions on rejection of CECs was not as evident with the Desal-5 DK membrane for which size exclusion seems to be the main removal mechanism.

### 3.2. Influence of Sodium Chloride Addition on the Rejection of PhACs, Pesticides, and PFAS with the RO Membrane

**Figure 4:**
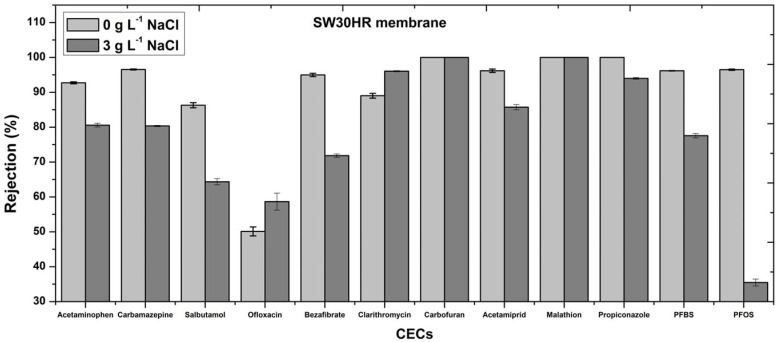
Effect of sodium chloride addition on the rejection of selected CECs by the RO membrane SW30HR.

Rejections of the twelve selected PhACs, pesticides, and PFAS by RO (SW30HR membrane) were studied in aqueous media with the addition of sodium chloride (concentration of 3 g L^−1^) at pH 7 (Figure 4). Increase in ionic strength of the feed solution might impact the membrane surface charge and influence the CECs rejection. Sodium chloride addition negatively affected the rejection of eight of the selected CECs compared to the sample without sodium chloride added. A decrease in rejection values of CECs with the SW30HR membrane was observed for all negatively charged CECs at pH 7, including carbamazepine, bezafibrate, acetamiprid, propiconazole, PFOS, and PFBS. Addition of inorganic compounds might decrease the membrane surface charge through chemical adsorption and electrostatic shielding. Since the SW30HR membrane is negatively charged at pH 7, addition of Na^+^ ions might suppress negative surface charge of the membrane [16]. Suppressed surface charge of SW30HR membrane decreases impact of electrostatic repulsion between negatively charged CECs and the RO membrane, resulting in lower rejection values compared to experiments without sodium chloride.

Decreased rejection values of positively charged acetaminophen and salbutamol at pH 7 by the SW30HR membrane were also observed. Based on the logK_o/w_ value, acetaminophen and salbutamol are considered hydrophilic. Addition of sodium chloride in a feed solution might induce dissociation of compounds at the surface of the membrane. Therefore, enhanced concentration of hydrophilic acetaminophen and salbutamol at the membrane surface could support increased membrane permeation of hydrophilic CECs, thus decreasing the rejection.

Increased rejection values of clarithromycin and ofloxacin by RO membrane were observed with addition of sodium chloride to the feed solution. The increased rejection for positively charged clarithromycin and ofloxacin, and decreased rejection of negative charged CECs, contributed to the general mechanistic differentiation from charge screening as dominant sodium chloride effect. Sodium chloride affected primarily membrane negative charge rather than CEC charge screening. Electrostatic attraction between the SW30HR membrane surface and positively charged clarithromycin at pH 7 decreases due to suppressed membrane surface charge, consequently slightly increasing rejection. Furthermore, apparent 100% rejection values of carbofuran and malathion were observed with and without sodium chloride addition. Since ofloxacin and malathion are neutral at pH 7, decrease in membrane surface charge would not impact the rejection. Furthermore, based on SW30HR MWCO (100 Da) and the general usage in desalination processes, it could be expected to reach higher rejection values concerning all tested CECs (CECs MM > SW30HR MWCO). However, the obtained results, both with and without sodium chloride added, showed relevance of other mechanisms besides size exclusion which negatively affected total rejection values obtained when using SW30HR membrane.

## 4. Conclusions

This study included the evaluation of NF and RO implementation for the removal of pharmaceuticals, pesticides, and PFAS (contaminants of emerging concern—CECs) from aqueous media at reported environmentally relevant concentrations, thereby addressing a critical gap in the current literature, which predominantly focuses on higher, non-environmental levels. Twelve compounds were selected based on their molecular descriptors (MM, pKa, and logK_o/w_) with focus on covering a wide range of molecular properties. Rejection values ranged from 42.07% to apparent 100% by selected membranes and were greatly influenced by operating pH, MM, and pKa value. These findings demonstrated a different impact of evaluated molecular descriptors, reinforcing previously reported studies with CEC-specific analysis and mechanistic clarification. Higher operating pH values increased CECs rejection values when using the SW30HR and NF270 membranes; however, the operating pH impact on CECs rejection when using the Desal-5 DK membrane was not as clear. MM greatly influenced CECs’ rejection values at all pH values, whereas logK_o/w_ values’ influence was rather minor. Electrostatic repulsion or electrostatic attraction strongly affected rejection values. Sodium chloride addition to the feed solution decreased rejection of eight selected CECs by the RO membrane. Size exclusion and electrostatic interactions were identified as the main removal mechanisms for selected PhACs, pesticides, and PFAS, with a lower influence of hydrophobic interactions. Future research could validate reported findings in more complex natural matrices where organic matter, colloids, and competitive sorption could modify CECs–membrane interactions. Additionally, sufficiently large amount of obtained data opens possibilities for exploring data-driven modeling approaches to correlate process descriptors (operating conditions, membrane properties, and solute molecular characteristics) with performance outputs, such as rejection, which could provide valuable insights.

## Figures and Tables

**Table 1:** Structure and properties of selected PhACs, pesticides, and PFAS.

CECs	Structure	MM (Da) ^a^	pKa ^a^	logK_o/w_ ^a^	Hydrophobicity ^b^
Pharmaceutically active compounds					
Clarithromycin	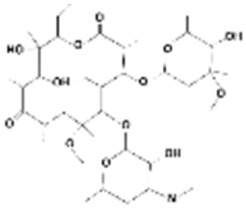	748.0	8.99	3.16	Hydrophobic
Ofloxacin	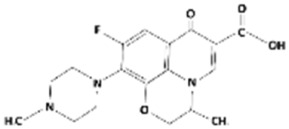	361.4	5.97–8.22	–0.39	Hydrophilic
Carbamazepine	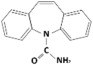	236.3	2.3	2.45	Hydrophobic
Acetaminophen	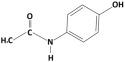	151.2	9.38/9.7	0.46	Hydrophilic
Salbutamol	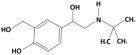	239.3	9.1–10.4	1.4 –0.64	Hydrophilic
Bezafibrate	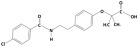	361.8	3.6	4.3	Hydrophobic
Pesticides					
Carbofuran	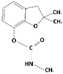	221.3	11.9	2.32	Hydrophobic
Acetamiprid	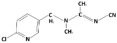	222.7	0.7	0.8	Hydrophilic
Malathion	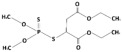	330.4	6.8	2.36	Hydrophobic
Propiconazole	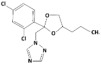	342.2	1.09	3.7	Hydrophobic
PFAS					
Perfluorooctane sulfonamide (PFOSA)	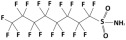	499.1	6.2	5.8	Hydrophobic
Perfluorooctane sulfonic acid (PFOS)	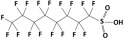	500.1	0.14	4.49	Hydrophobic
Perfluorobutanoic acid (PFBA)	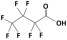	214.0	0.08	2.2	Hydrophobic
Perfluorobutane sulfonic acid (PFBS)	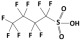	300.1	–3.31	1.82	Hydrophilic

^a^ Pubchem, Chemspider; ^b^ determined based on logK_o/w_ value (logK_o/w_ > 2 hydrophobic, logK_o/w_ < 2 hydrophilic) [29].

**Table 2:** Characteristics of the selected NF and RO membranes.

Commercial Name	SW30HR	Desal-5 DK	NF270
Type	RO	NF	NF
Manufacturer	FilmTec™	SUEZ	FilmTec™
Material ^a^	TFC polyamide	TFC polyamide	TFC polyamide
MWCO (Da) ^a^	100	150–300	400
pH range ^a^	2–11	2–10	2–11
pKa	4.0 ^d^	4.1 ^c^	3.3/5.0 ^b^
Surface charge at pH 4 ^e^	neutral	neutral	neutral
Surface charge at pH 7 ^e^	negative	negative	negative
Surface charge at pH 10 ^e^	negative	negative	negative

^a^ According to the manufacturers’ specifications; ^b^ [31,32]; ^c^ [33]; ^d^ [32]; ^e^ determined based on pKa value (pH = pKa, neutral; pH > pKa, negative).

**Table 3:** Summary of the potential removal mechanisms of PhACs, pesticides, and PFAS by SW30HR, Desal-5 DK, and NF270 membranes at pH 4, pH 7, and pH 10.

pH	4			7			10		
Removal Mechanisms	Size Exclusion	Electrostatic Interactions	Hydrophobicity	Size Exclusion	Electrostatic Interactions	Hydrophobicity	Size Exclusion	Electrostatic Interactions	Hydrophobicity
Acetaminophen	+	−	−	+	+	−	+	+	−
Carbamazepine	+	−	+	+	+	−	+	+	−
Salbutamol	+	−	−	+	+	−	+	−	−
Ofloxacin	+	−	−	+	−	−	+	+	−
Bezafibrate	+	−	+	+	+	−	+	+	−
Clarithromycin	+	−	−	+	−	−	+	−	−
Carbofuran	+	−	+	+	−	−	X	X	X
Acetamiprid	+	−	−	+	+	−	+	+	−
Malathion	+	−	+	+	−	−	X	X	X
Propiconazole	+	−	+	+	+	−	+	+	−
PFBS	+	−	−	+	+	−	+	+	−
PFOS	+	−	+	+	+	−	+	+	+

+ observed impact on the rejection; − impact on rejection not observed; X—compound was not detected in feed, permeate, or retentate.

## Data Availability

Data supporting reported results can be found at the following link: https://doi.org/10.5281/zenodo.15790703 (accessed on 2 November 2025).

## Supplementary Materials

The following supporting information can be downloaded at: https://www.mdpi.com/article/10.3390/membranes15120358/s1. Figure S1: Rejection of selected CECs at three pH values by SW30HR, Desal-5 DK, and NF270 membranes; Method: UHPLC-MS/MS.; Table S1: Gradient profile—time, percentage of mobile phases and flow rate; Table S2: MS/MS parameters; Table S3: Recovery and RSD of selected CECs.

## Abbreviations

The following abbreviations are used in this manuscript:

CECs	Contaminants of emerging concern
HLB	Hydrophilic–lipophilic balance
MeOH	Methanol
MM	Molecular mass
MWCO	Molecular weight cut off
NF	Nanofiltration
PFAS	Poly- and perfluoroalkyl substances
PFBA	Perfluorobutanoic acid
PFBS	Perfluorobutane sulfonic acid
PFOA	Perfluorooctanoate
PFOS	Perfluorooctane sulfonic acid
PFOSA	Perfluorooctane sulfonamide
PhACs	Pharmaceutically active compounds
RO	Reverse osmosis
SPE	Solid phase extraction
TFC	Thin-film composite
UHPLC-MS/MS	High performance liquid chromatography coupled with triple quadrupole mass spectrometry
