# Supplementary material for: Pharmaceuticals, Pesticides, and Poly- and Perfluoroalkyl Substances at Surface Water Occurrence Levels—Impact of Compound Specific Physicochemical Properties on Nanofiltration and Reverse Osmosis Processes
Source: Membranes (Basel). 2025 Nov 27;15(12):358. doi: 10.3390/membranes15120358 (PMC12734873; doi:10.3390/membranes15120358)
Supplement: Supplementary file 1 [file membranes-15-00358-s001.zip › membranes-3995197-supplementary.pdf]

Supplementary material

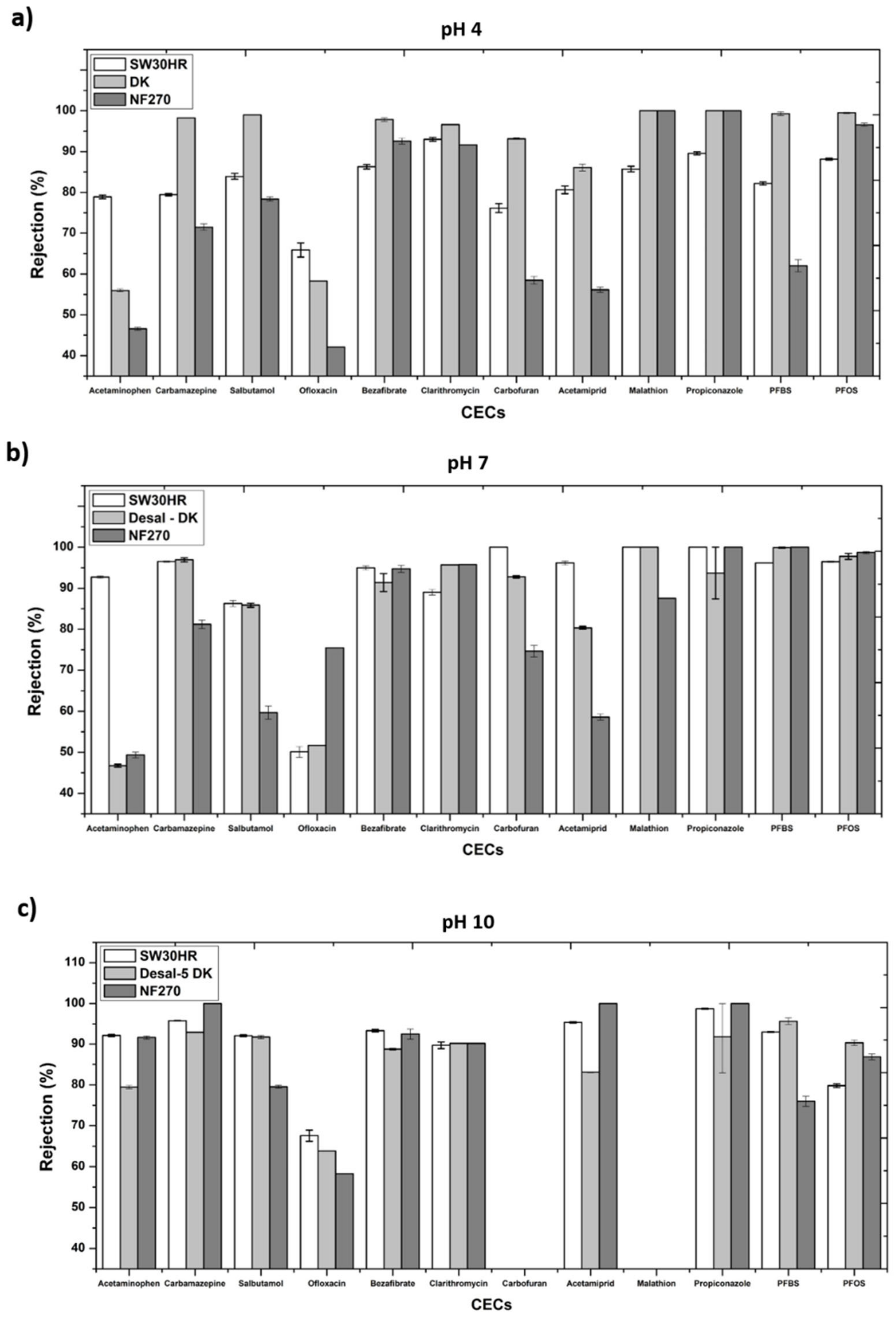

Figure S1 Rejection of selected CECs at three pH values by SW30HR, Desal-5 DK and NF270 membranes with error bars

UHPLC-MS/MS

The instrumental method used was previously described in Rakić et al. (2023). ZORBAX Eclipse Plus C18, 100x2.1 mm i.d., 1.8 µm column (Agilent Technologies, USA) with a flow rate 400 µL/min and temperature maintained at 30 °C was used for the separation of analyzed compounds. Injection volume was 10 µL, whereas the mobile phase consisted of eluent A containing water/formic acid (99.9:0.1, v/v) and 5 mM ammonium acetate and eluent B (MeOH). The gradient profile is given in Table S1, total time of the run was 13 min. Each sample was injected twice and a mean value and standard deviations were calculated from obtained values. Triple quadrupole mass spectrometer (MS/MS) TSQ Vantage (Thermo Fisher Scientific, Waltham, Massachusetts, USA) equipped with heated-electrospray ionization probe (HESI-II, Thermo Scientific, Waltham, Massachusetts, USA) was used for CECs quantification. Spray voltage, vaporizer temperature, and capillary temperature of the ion source were set at 3.4 kV, 250°C, and 270°C, respectively. Sheath gas pressure and auxiliary gas pressure of the ion source were set at 40 (arbitrary units) and 10 (arbitrary units), respectively. Calibration curves were generated using linear regression analysis. Calibration was accepted if R2>0.9900 over the established concentration range from 1 to 400 ng/L. Calibration solutions were prepared in the first gradient phase used for chromatographic separation (H_2O:MeOH = 95:5, v/v) and stock solution was prepared in MeOH. During the preparation of calibration solutions, an equal amount of stock solution and solution of the first gradient of the mobile phase were used (50 µL + 950 µL, respectively).

Table S1 Gradient profile - time, percentage of mobile phases and flow rate.

Time (min)	Mobile phase A (%)	Mobile phase B (%)	Flow (µL/min)
0.00	95	5	400
0.30	95	5	400
5.00	70	60	400
6.50	35	65	400
8.50	0	100	400
11.00	0	100	400
11.50	95	5	400

13.00	95	5	400

MS/MS parameters, including retention time, parent mass, product mass, collision energy, polarity, start and end time, as well as isotropically labelled standard for each selected CECs are shown in Table S2. Recovery and recovery standard deviation (RSD) are shown in Table S3.

Table S2 MS/MS parameters

CECs	Retention time (min)	Parent mass, m/z	Product mass 1/ Collision energy (eV)	Product mass 2/ Collision energy (eV)	Polarity (+/-)	Start time (min)	End time (min)	Isotopically labelled standard
Clarithromycin	8.25	748.30	157.96/28	590.21/17	+	7.2	10.0	d10_carbamazepine
Ofloxacin	6.40	362.14	261.03/26	318.08/18	+	5.5	7.0	d8_ofloxacin
Carbamazepine	8.01	237.10	192.10/24	194.10/19	+	7.5	8.7	d10_carbamazepine
Acetaminophen	3.62	152.10	65.10/31	110.10/16	+	3.0	4.8	d7-sotalol
Salbutamol	3.69	240.16	148.07/18	222.11/10	+	3.0	4.6	d7-sotalol
Bezafibrate	8.50	360.10	153.97/30	273.99/20	-	8.0	9.2	d6_bezafibrate
Carbofuran	7.84	222.10	123.06/22	165.11/12	+	7.4	9.0	d3_carbofuran
Acetamiprid	7.09	223.07	73.02/53	126.00/21	+	6.5	8.0	d3_acetamiprid
Malathion	8.62	331.00	124.99/27	284.94/20	+	8.0	9.5	d7_malathion
Propiconazole	9.13	343.06	158.93/34	160.00/33	+	8.5	10	d7_malathion
PFOSA	9.22	497.79	78.00/39	258.99/24	-	8.6	10.0	M6PFDA
PFOS	8.82	498.77	80.01/48	98.99/45	-	8.2	9.5	M8PFOA
PFBA	5.59	212.99	119.00/8	169.04/11	-	5.2	6.5	MPFBA
PFBS	7.70	298.93	80.02/40	99.01/31	-	7.0	8.5	M5PFPeA

Table S3 Recovery and RSD of selected CECs

CECs	Recovery and precision (%)	RSD (%)
Clarithromycin	105.51	10.23%
Ofloxacin	46.03	3.16%
Carbamazepine	100.96	4.85%
Acetaminophen	98.43	10.76%
Salbutamol	79.62	6.78%
Bezafibrate	73.87	5.37%
Carbofuran	85.57	10.10%
Acetamiprid	91.17	11.41%
Malathion	71.57	6.97%
Propiconazole	62.42	6.83%
PFOSA	7.36	3.53%
PFOS	102.48	4.17%
PFBA	20.92	1.81%
PFBS	111.28	6.09%

References

Rakić D., Antić I., Živančev J., Buljovčić M., Šereš Z., Đurišić-Mladenović N. (2023): Solid-phase extraction as promising sample preparation method for compound of emerging concerns analysis, Analecta Technica Szegedinensia, 17(4), 16-24.
